# Osteitis Pubis in Athletes: A Literature Review of Current Surgical Treatment

**DOI:** 10.7759/cureus.22976

**Published:** 2022-03-08

**Authors:** Vasileios Athanasiou, Anastasia Ampariotou, Ioanna Lianou, George Sinos, Antonis Kouzelis, John Gliatis

**Affiliations:** 1 Orthopaedics and Traumatology, University General Hospital of Patras, Patras, GRC; 2 Orthopaedics and Traumatology, Patras University Hospital, Patras, GRC

**Keywords:** pubic symphysectomy, pubalgia, sports overuse injuries, groin pain, osteitis pubis

## Abstract

Osteitis pubis (OP) is a self-limiting, noninfectious inflammatory disease of the pubic symphysis and the surrounding soft tissues that usually improves with activity modification and targeted conservative treatment. Surgical treatment is required for a limited number of patients. This study aims to investigate the current literature on the surgical treatment of OP in athletes.

A systematic review was conducted on two databases (MEDLINE/PubMed and Google Scholar) from 2000 to 2021. The inclusion criteria were adult patients with athletic OP who underwent surgical treatment and studies published in English. The exclusion criteria included pregnancy, infection OP, or postoperative complications related to other surgical interventions, such as urological or gynecological complications.

Fifty-one surgically treated cases have been reported in eight studies, which included short-term, mid-term, and long-term studies ranging from one patient to 23 patients. The surgical treatment methods were as follows: (a) pubic symphysis arthrodesis, (b) open or endoscopic pubic symphysectomy, (c) wedge resection of the pubic symphysis, and (d) polypropylene mesh placed into the preperitoneal retropubic space endoscopically.

The main indication for surgical intervention was failure of conservative measures and long-lasting pain, disability, and inability to participate in athletic activities. Wedge resection of the pubic symphysis has been the less preferred surgical treatment in the recently published literature. The most common surgical method of treatment of OP in athletes, which entailed the existence of posterior stability of the sacroiliac joint, in the current literature is open pubic symphysis curettage. Recently, there has been a tendency for pubic symphysis curettage to be performed endoscopically.

## Introduction and background

Osteitis pubis (OP) in athletes is an idiopathic inflammatory condition that affects the pubic symphysis and the surrounding soft tissues and is caused by overuse or trauma [1-7]. It was first documented as a complication after suprapubic surgery by Beer in 1924, but it was later renamed athletic OP by Spinelly in 1932 [5]. OP is more common in high-level athletes, who train intensively, such as soccer, rugby, Australian Rules football, distance running, and ice hockey players [6,7]. It is characterized by pain in the pubic symphysis that worsens with physical activity. The prevalence of athletic OP has recently been observed to range from 0.5% to 8%. However, in kicking sports, mainly male soccer players sustain significantly more injuries, at a frequency ranging from 10% to 18% per year [2,4,6,7]. OP is a self-limiting disease that improves with activity modification and individualized conservative treatment, while surgical treatment is required for about 5% to 10% of patients. However, not all athletes are eligible for conservative treatment due to difficulties in pain management and the long or unpredictable time frame of conservative treatment [6,7]. The current surgical options are as follows: (a) open-wedge symphyseal resection, (b) symphyseal fusion, (c) operations to strengthen or restore abdominal or pelvic floor musculature, and (d) open or endoscopic symphysis curettage. There is no time frame before the surgical treatment of OP in athletes, and it is not quite clear what could be the best surgical technique with a view to full sports activity participation. This study aims to investigate the current literature on the surgical treatment of OP in athletes. More precisely, this review aims to answer the following questions: (a) which is the most preferred surgical technique in the last two decades, (b) what are the perioperative complications, and (c) what is the time span required to return to full activity and what the percentage of athletes who returned to full activity after the surgical treatment?

## Review

Search strategies and inclusion criteria

A systematic review was conducted on two databases (MEDLINE/PubMed and Scholar Google) using the keywords “osteitis pubis in athletes,” “groin pain in athletes,” “pubalgia in athletes,” and “pubic symphysectomy” in the English language between January 1, 2000, and December 30, 2021. The inclusion criteria were adult patients with athletic OP who underwent surgical treatment and studies published in English. The exclusion criteria included pregnancy, infection OP, or postoperative complications related to other surgical interventions, such as urological or gynecological complications. Abstracts were screened by two reviewers (IL and GS) independently.

Results

**Figure 1:**
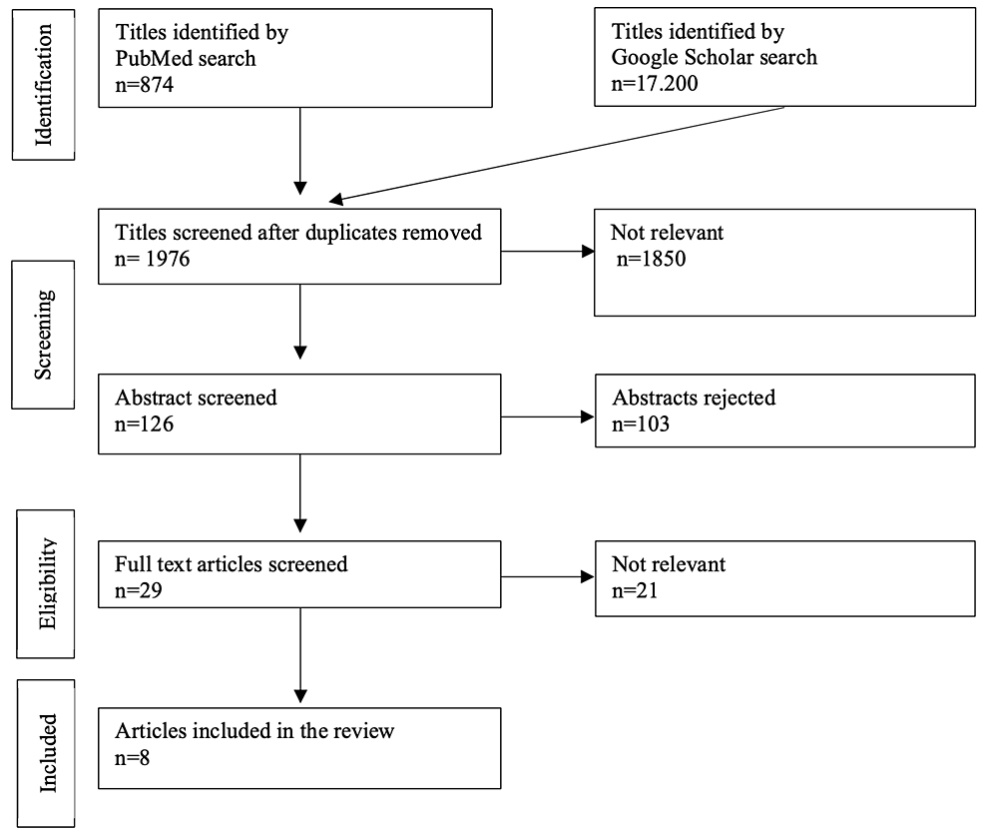
PRISMA literature search methodology PRISMA: Preferred Reporting Items for Systematic Reviews and Meta-Analyses

Data were extracted as follows: our literature review revealed 1976 studies, of which 126 studies were eligible for abstract review and 29 studies for full-text review. There were no comparative studies; all were case reports or case series with low (IV) levels of evidence. Finally, eight studies [6,8-14] were found to be eligible for inclusion in our review (Figure 1).

Fifty-one (51) surgically treated cases were published in eight papers, which included short-term, mid-term, and long-term studies ranging from one case to a series of cases (Table 1). Eight of the cases were associated with femoroacetabular impingement (FAI). There were 15 Australian Rules football players, 12 soccer-football players, nine rugby players, one bandy player, one cross-country skier, one field hockey player, two cases referred as trauma, and seven athletes with no sports referred. There were 44 men and seven women, with a mean age of presentation of 27.04 years (range: 19.9-56.7 years). Twenty-five athletes underwent open curettage, eight athletes underwent endoscopic pubic symphysectomy, seven athletes underwent arthrodesis, four athletes underwent a combination of an osteotomy and curettage, two athletes underwent wedge resection, and a polypropylene mesh was placed into the preperitoneal retropubic space endoscopically in five athletes. The mean time of conservative treatment before surgical treatment ranged from 2 months to 48 months.

**Table 1:** Descriptive table with the number of patients, surgical treatment, and results across all included studies [6,8-14]

Author, year, and reference number	Patient number	Patient age	Sports	Time of conservative treatment	Surgical treatment	Complications	Return to sports activity	Results
Williams et al. (2000) [8]	7	24.7 years (range: 21–29 years)	Rugby players	13 months (range: 13–48 months)	Arthrodesis (vertical instability)	One hemospermia for six weeks and one intermittent scrotal swelling during exercise for six months	6.6 months (range: 5–9 months)	All patients were free of symptoms at a mean follow-up of 52 months (range: 10 months–12 years)
Mulhall et al. (2002) [9]	2	25 and 26 years	Professional soccer players	One and 1.5 years	Open curettage + methylprednisolone (40 mg) and 0.5% bupivacaine injection	No complications reported	Within six months	Both patients were free of symptoms
Paajanen et al. (2005) [10]	5	27.5 years (range: 21–35 years)	Three soccer players, one bandy player, and one cross-country skier	Several months	Polypropylene mesh 10 × 15 cm endoscopically into the preperitoneal retropubic space	No complications reported	Gradually resumed in all five patients after four to eight weeks of convalescence	All patients were free of symptoms one month and one year postoperatively
Mehin et al. (2006) [11]	2/10 trauma	40 years (range: 20–55 years)	Not reported	Four years (range: 8–10 months)	Five wedge resections and five arthrodesis	Four had persistent discomfort	Not reported	1/5 with wedge resection and 3/5 with arthrodesis were considered failure
Radic et al. (2008) [6]	23	27.04 years (range: 19.9–56.7 years)	15 Australian Rules football, five soccer, two rugby, and one hockey players	13.2 months (range: 2–36 months)	Open curettage	No significant postoperative complications	21 patients returned to pain-free running by 3.14 months (range: 1.5–6 months), 17 to training by 4.44 months (range: 2.5–7 months), and 16 to full activity by 5.63 months (range: 2.5–12 months)	One did not regain full activity, two did not reach pain-free running, and four were unable to return to full training
Hechtman et al. (2010) [12]	4	22.4 years (range: 20–26 years)	Two professional and two collegiate football players	10 months (range: 6–13 months)	Osteotomy and curettage	No complications reported	Three months (range; 2–8 months)	All patients were free of symptoms
Matsuda (2010) [13]	1	31-year-old female	Athletic patients	Recalcitrant osteitis pubis	Endoscopic pubic symphysectomy	No complications reported	12 months following patient-reported high satisfaction	Patient was pain-free
Matsuda et al. (2015) [14]	7	Mean age of 33 years	Athletic patients	Not reported	Endoscopic pubic symphysectomy	Two male patients had postoperative transient scrotal swelling, and one patient has persistent pain	Not reported	One review arthrodesis; the mean patient satisfaction rating was 8.3 (range: 3–10)
	(51)							

Statistical analysis

Time A (Conservative Treatment)

We perform analysis of variance (ANOVA) with the use of summary statistics (N, mean, and SD). SD is computed as the range divided by 4, when available. Post hoc pairwise comparisons are held via the Tukey HSD test.

The overall p-value is 0.0001. More specifically, the post hoc pairwise comparisons are as follows: arthrodesis versus open curettage: p = 0.9998 (NS), arthrodesis versus osteotomy and curettage: p = 0.9473 (NS), arthrodesis versus wedge resection: p = 0.0001, open curettage versus osteotomy and curettage: p = 0.9052 (NS), open curettage versus wedge resection: p < 0.0001, and osteotomy and curettage versus wedge resection: p = 0.0001.

Time A (Return to Sports Activity)

We perform analysis of variance (ANOVA) with the use of summary statistics (N, mean, and SD). SD is computed as the range divided by 4, when available. The overall p-value is 0.0009, in arthrodesis versus osteotomy and curettage.

Complications

Comparing 2/7 complications of arthrodesis to zero, p = 0.3898 (NS). Comparing 3/8 complications of endoscopic pubic symphysectomy to zero, p = 0.2983 (NS).

Discussion

The etiology of OP is not completely understood [15]. However, it is commonly believed that the cause of athletic OP is the presence of overuse stress injury. Moreover, there are two relevant theories, chronic muscle imbalance theory and the increased compensatory motion of the pubic symphysis theory. According to the first theory, many muscles are inserted into and originate from the anterior pelvis ring antagonists, such as the adductor muscle complex and rectus abdominis. The chronic imbalance between these muscles causes abnormal forces across the pubic symphysis affecting the joint’s biomechanics, resulting in bone stress injury and cartilage degeneration. According to the second theory, pubic symphysis increases motion to compensate for the less movement of another part of the chain movement, such as the femoroacetabular syndrome [4].

The articular surfaces of the pubic symphysis have an oval shape, with a mean length ranging from 30 mm to 35 mm and a mean width ranging from 10 mm to 12 mm. The articular cartilage thickness ranges from 1 mm to 3 mm. The fibrocartilaginous disc between the articular surfaces of the pubic bones has a wedge or Y-shape with the apex directed posteriorly. Pubic symphysis has four ligaments: the superior, inferior, anterior, and posterior pubic ligaments [16].

In an in vivo study of the mobility of the pubic symphysis, Walheim and Selvik reported translations up to 2 mm and rotations up to 3 degrees [17]. Recently, Giannoudis reported normal movement of the pubic symphysis in men up to 0.5 mm, compared to women of up to 1.5 mm. In contrast, up to 3 mm might occur after more than two pregnancies [18].

Athletes with OP complain of pain located in the pubic symphysis and the medial zone of the groin, which radiates to the adductors, suprapubic, and lumbar regions and aggravates with athletic activities depending on the degree of OP stage. Therefore, Rodriguez et al. classified athletic OP into four stages (Table 2) based on a small number of patients and clinical examination and diagnostic characteristics [7,19-21].

**Table 2:** Rodriguez et al.’s classification based on MRI and clinical findings [19]

Stage	Side of pain	Site of pain	Characteristics of pain
1	Unilateral, dominant	Inguinal, with radiation to adductors	Pain alleviation after warm-up, pain exacerbation after training
2	Bilateral	Inguinal and adductors	Pain exacerbation after training
3	Bilateral	Groin and adductor, suprapubic, and abdominal regions	During training, kicking, sprinting, and turning; cannot achieve training goals, forced to withdraw
4	Generalized	Generalized, with radiation to the lumbar region	Walking, getting up, straining at stool, simple activities of daily living

The diagnosis of athletic OP is challenging due to anatomical complexity and overlapping symptoms with athletic pubalgia and athletic hernia. Verrall et al. proposed three provocation tests: the single adductor test, the squeeze test, and the bilateral adductor test [20,22]. As diagnostic tools, some authors suggest local anesthetic injection with or without corticosteroids [7,23,24]. Restricted range of hip motion and sacroiliac joint dysfunction may associate with athletic OP [7,25]. Saito et al. found 67.8% radiographic evidence of OP in 28 soccer players with asymptomatic femoroacetabular impingement syndrome; magnetic resonance imaging (MRI) showed positive marrow edema (BME) in 35.7% of these cases, which significantly improved after hip arthroscopy [26].

Histological studies of the samples obtained after the surgery of OP demonstrated woven immature bone with neovascularization, osteoblasts, fibroblasts, degenerative cartilage free of inflammatory cells, or signs of osteonecrosis [4,22,27].

In the absence of pathognomonic imaging, radiographs, MRI, and triple-phase scintigraphy along with a comprehensive clinical examination and the patients’ medical history can confirm or exclude other sources of pain [7,21,28]. In an early stage, radiographs may be normal but can confirm or rule out other sources of pelvic pain. In the chronic phase, osteolytic, osteosclerotic, and widening changes of the pubic symphysis may appear. Flamingo views with more than 2 mm of vertical translation are defined as radiographic instability of pubic symphysis [7,2]. Magnetic resonance imaging (MRI) can be helpful in the diagnosis of OP. MRI demonstrates bone edema in the acute phase, but it may not be present in the chronic phase [21]. In 2012, Krüger proposed a classification of OP (Table 3) based on MRI and clinical findings [29]. In 2017, Gaudino et al. also proposed a new MRI classification (Table 4) based on information of both severity and prognosis of OP [30]. Triple-phase scintigraphy is also an assisting tool for the investigation of OP. However, a poor correlation between bone scan findings and the location and duration of symptoms has been reported [21].

**Table 3:** Krüger’s classification based on MRI and clinical findings [29]

Stadium	MRI signs	Pain	Duration of symptoms
Ι	Bone marrow edema at one side or bilateral at the pubic bone	Inguinal/adductor muscles	Symptoms lasting up to three months
ΙΙ	Edema at soft tissue around the symphyseal joint or at the muscle junction	Inguinal/adductor muscles	Symptoms lasting up to six months
ΙΙΙ	Edema/fluid in the muscles located around the symphyseal cleft joint with or without secondary cleft sign	Complex/pelvic muscle complex	Symptoms lasting up to 12 months

**Table 4:** Gaudino’s classification based on information of both severity and prognosis [30]

Grade	MRI findings	Complete recovery
I	Bone marrow edema + highest mean normalized STIR SI < 3 +/- periarticular edema	100%
II	Bone marrow edema + highest mean normalized STIR SI < 3 periarticular edema + edema in the muscles around the symphyseal joint	50%
III	Bone marrow edema + highest mean normalized STIR SI ≥ 3 +/- one of the following: periarticular edema or edema in the muscles around the symphyseal joint	30%
IV	Bone marrow edema + highest mean normalized STIR SI ≥ 3 + periarticular edema + edema in the muscles around the symphyseal joint	20%

OP in athletes is self-limiting but may last from several months to years. The role of steroids, PRP, or prolotherapy injections remains controversial. Conservative treatment can be effective initially [31]. Nevertheless, approximately 5%-10% of athletes require surgical treatment. There is no fixed time frame of when conservative treatment is considered a failure, yet generally, six months of treatment without relief of symptoms is an indication for operative intervention [7,21]. Surgical options include endoscopic or open symphysis curettage, symphyseal fusion with or without bone graft, open-wedge resection of the symphysis with or without arthrodesis, and procedures to reinforce or repair abdominal or pelvic floor musculature. Surgical procedures can be associated with the release of the adductor tendons or with adductor enthesis repair [21,24,32].

The wedge resection of symphysis pubis for the treatment of OP was first described by Schnute in 1961 and has been recommended as a safe and reliable method for patients who do not respond to nonoperative treatment. It showed promising results provided the existence of sacroiliac joint stability [29,32]. However, a long period of return to full activity has been reported at an average of 14 months postoperatively [21]. Our study showed only two cases that underwent wedge resection of the symphysis pubis.

Symphysis pubis fusion has been reported with a percentage of postoperative complications of up to 25% [21]. Based on the flamingo views, Williams et al. reported seven professional male rugby players with pubic symphysis instability who underwent arthrodesis with plate and autologous tricortical iliac crest bone graft. Return to full activity was reported at a mean period of 6.6 months (range: 5-9 months). No graft site complications have been reported at a follow-up ranging from 10 months to 12 years despite one transient hemospermia for six weeks and one recurrent intermittent scrotal swelling [8].

Paajanen et al. reported a novel method in five elite-level male athletes with chronic groin pain associated with OP where a polypropylene mesh was placed in the retropubic preperitoneal space endoscopically. Full activity was allowed after four weeks. No postoperative complications were referred within a one-year follow-up [10]. This surgical technique shows good results although in a small number of athletes.

The majority of the patients (49%) have been treated by open pubic symphysis curettage. Radic et al. used a 2 mm drill with three passes into each pubic bone as well [6]. They reported that 69.5% of the patients return to full activity by 5.63 months. Overall, 78% of the patients referred that their symptoms were better or much better than those they had preoperatively. No significant perioperative or postoperative complications were reported. Mulhall et al. reported nonspecific postoperative complications [9].

Recently, there has been a tendency for the pubic symphysis curettage to be endoscopic. Gupta et al. described an endoscopic technique for pubic symphysectomy for the treatment of recalcitrant osteitis pubis [33]. In a multicenter retrospective case series of seven consecutive adult patients who suffered from OP associated with FAI, Matsuda et al. reported that two patients had postoperative transient scrotal swelling, and one patient, due to the continued pain, underwent pubic symphyseal arthrodesis [14].

Our current systematic literature review shows that wedge resection of the pubic symphysis is less preferred nowadays. The most common surgical technique is open symphysis resection, with 30% of the athletes not being able to regain full activity. Paajanen et al. reported good results regarding the perioperative complications and the time period to return to full activity with a polypropylene mesh placed in the retropubic preperitoneal space endoscopically [10]. However, in a small number of athletes, Williams et al. reported good long-term results in pubic symphysis instability treated with plate and autologous tricortical iliac crest bone graft [8]. Regarding the pubic symphysis curettage, there has been a tendency to be performed endoscopically [10,13,14].

Recently, an association of OP with FAI has been reported. For example, Saito et al. reported that in 28 soccer players with symptomatic FAI, 19 (67.8%) patients demonstrated radiographic evidence of OP and 10 (35.7%) bone marrow edema (BME) on MRI [26].

## Conclusions

The surgical treatment of OP in athletes remains controversial. Nowadays, wedge resection of the pubic symphysis is less preferred. The most common surgical method for the treatment of OP in athletes is open pubic symphysis curettage. Recently, there also has been a tendency for pubic symphysis curettage to be performed endoscopically. However, further research is needed to clarify the superiority of one surgical treatment over the other and which is the most promising therapeutic approach for athletes with OP.
